# Dyskeratosis congenita and the DNA damage response

**DOI:** 10.1111/j.1365-2141.2011.08679.x

**Published:** 2011-06

**Authors:** Michael Kirwan, Richard Beswick, Amanda J Walne, Upal Hossain, Colin Casimir, Tom Vulliamy, Inderjeet Dokal

**Affiliations:** 1 Centre for Paediatrics, Barts and The London School of Medicine and Dentistry, Queen Mary University of London, Barts and The London Children's HospitalLondon, UK; 2 School of Health & Social Sciences, Middlesex UniversityLondon, UK

**Keywords:** cell cycle, DNA damage, dyskeratosis congenita, lymphocytes, telomeres

## Abstract

Dyskeratosis congenita (DC) is a heterogeneous bone marrow failure disorder with known mutations in components of telomerase and telomere shelterin. Recent work in a mouse model with a dyskerin mutation has implicated an increased DNA damage response as part of the cellular pathology, while mouse models with *Terc* and *Tert* mutations displayed a normal response. To clarify how these contradictory results might apply to DC pathology in humans, we studied the cellular phenotype in primary cells from DC patients of several genetic subtypes, focussing on T lymphocytes to remain close to the haematopoietic system. We observed novel cell cycle abnormalities in conjunction with impaired growth and an increase in apoptosis. Using flow cytometry and confocal microscopy we examined induction of the DNA damage proteins γ-H2AX and 53BP1 and the cell cycle protein TP53 (p53). We found an increase in damage foci at telomeres in lymphocytes and an increase in the basal level of DNA damage in fibroblasts, but crucially no increased response to DNA damaging agents in either cell type. As the response to induced DNA damage was normal and levels of global DNA damage were inconsistent between cell types, DNA damage may contribute differently to the pathology in different tissues.

Dyskeratosis congenita (DC) is an inherited disorder characterized by muco-cutaneous abnormalities consistent with a premature ageing phenotype, bone marrow failure and a predisposition to cancer (Dokal, 2001). As well as serving as a model of ageing in humans it is also a model disease for telomere dysfunction as it is the first disease that is thought to be caused by premature telomere shortening (Calado & Young, 2009; Bessler *et al*, 2010). Seven genetic subtypes have been described, five of which have genetic lesions in components (dyskerin, TERC, TERT, NOP10 and NHP2) belonging to the telomerase holoenzyme (Heiss *et al*, 1998; Vulliamy *et al*, 2001, 2008; Armanios *et al*, 2005; Walne *et al*, 2007), which is responsible for maintaining telomere length in certain tissues, and one of which (TIN2) has a lesion in the telomere shelterin complex (Savage *et al*, 2008), which is responsible for maintaining the structural integrity of the telomere (de Lange, 2005).

While the disease was first thought to be due to defective ribosome biogenesis (Heiss *et al*, 1998; Luzzatto & Karadimitris, 1998) data published in the intervening years has heavily implicated premature telomere shortening as the primary cause of the pathology. Indeed DC patients, as a group, have very short telomeres (Mitchell *et al*, 1999; Alter *et al*, 2007) and the clinical phenotype is consistent with premature loss of cells in tissues with high turnover such as the skin, gut and bone marrow.

Recent studies have suggested that cells with DC-relevant mutations have an increased DNA damage response that has previously been overlooked and that this may play an important role in disease pathology (Gu *et al*, 2008). However, the current DNA damage studies have been carried out only in a single cell type (fibroblasts) with much of the work having been done in mouse models (Gu *et al*, 2008; Vidal-Cardenas & Greider, 2010). The only analysis of characterized DC patient cells was in a single family with a *TERC* mutation (Westin *et al*, 2010). We therefore wanted to determine how prominent this potential mechanism might be in DC pathophysiology.

In this study we analysed primary human tissue exclusively, with the aim of avoiding, wherever possible, artefacts from transformation or genetic modification and potential differences between human cells and animal models. We obtained cells from patients with mutations in the telomerase genes *DKC1* (the gene encoding dyskerin, a telomerase structural nucleoprotein), *TERC* (the telomerase RNA component) and *TERT* (the telomerase reverse transcriptase), which collectively constitute the majority of genetically characterized DC cases. As bone marrow failure is the cause of mortality in approximately 80% of DC cases (Kirwan & Dokal, 2009) we focussed the majority of our analyses on T lymphocytes as these are cells of the haematopoietic system. We also studied fibroblasts in order to compare our data with the published literature.

While our data confirm that cell growth and survival are impaired and that apoptosis is increased, we also uncovered novel cell cycle abnormalities with fewer DC cells in the dividing S and G_2_ phases and more in the senescent/quiescent G_0_ phase. Extensive analysis of the DNA damage response revealed increased basal levels of DNA damage in fibroblasts and increased damage at telomeres in lymphocytes but crucially, the response to freshly induced DNA damage was not increased. We therefore concluded that, while DNA damage is increased – consistent with the accumulation of short or dysfunctional telomeres – this is inconsistent between cell types and we found no evidence that increased response to induced DNA damage is responsible for the cellular or clinical phenotype.

## Materials and methods

### Patient T lymphocyte acquisition and tissue culture

Blood samples were obtained from patients and unaffected control volunteers at Barts and The London Children's Hospital, London with informed, written consent and research ethics committee approval. T lymphocytes were separated using lymphocyte separation medium (Lonza, Wokingham, UK) and selected for by RosetteSep antibody cocktail (Stem Cell Technologies, Grenoble, France) according to the manufacturer's instructions, washed twice with phosphate buffered saline (PBS) and resuspended at 1·0 × 10^6^ cells/ml in RPMI-1640 medium + 10% fetal calf serum (FCS; Invitrogen, Paisley, UK). Purity of the isolated T lymphocytes was determined by flow cytometry using a monoclonal anti-human CD3ε mouse IgG conjugated to allophycocyanin (R&D Systems, Abingdon, UK) according to the manufacturer's instructions. Mitosis was stimulated by the addition of 10 μg/ml of phytohaemagglutinin (PHA; Roche Applied Science, Burgess Hill, UK). After 48 h the medium was replaced with fresh RPMI containing 10 units/ml of recombinant human interleukin 2 (Invitrogen). Cells were counted on a NucleoCounter YC-100 automated cell counter (ChaemoMetec, Allerod, Norway). Fibroblasts were cultured in RPMI-1640 medium + 10% FCS. All three DC fibroblasts contained *DKC1* mutations (c.106T>G/p.Phe36Val, c.361A>G/p.Ser121Gly and c.1058C>T/p.Ala353Val).

### Telomere length measurement

Telomere lengths were measured by Southern blot of genomic DNA from peripheral blood leucocytes using a subtelomeric probe, pTelBam8, as previously described (Notaro *et al*, 1997).

### Cell cycle analysis

All flow cytometry was conducted using an LSRII four laser flow cytometer (Becton Dickinson, UK, Ltd. (BD), Oxford, UK). Apoptosis levels were determined using the Vibrant Apoptosis Assay Kit#3 (Invitrogen) according to the manufacturer's instructions before flow cytometry. Briefly, this involved staining of nuclear DNA with propidium iodide (PI) and co-staining of the apoptotic membrane marker phosphatidylserine with a fluorescent annexin V conjugate.

G_0_/G_1_, S and G_2_M populations were determined using standard methods. 0·2 × 10^6^ cells were fixed overnight in 70% ethanol, washed twice in PBS and resuspended in 300 μl PBS with 50 μg/ml PI and 200 μg/ml RNAse A. Cells were incubated at 37°C for 30 min before being analysed on the flow cytometer. Cells were pulse-width gated to exclude doublets.

G_0_ and G_1_ populations were determined using a method modified from Ladd *et al* (1997). Briefly, 0·2 × 10^6^ cells were washed twice in PBS and resuspended in 300 μl PBS with 10 μg/ml Hoescht 33342 and 0·5 μg/ml Pyronin Y. Cells were incubated at 37°C for 45 min before being analysed on the flow cytometer. Cells were pulse-width gated to exclude doublets.

### DNA damage-associated γ-H2AX and TP53 (p53) analysis

To assay levels of γ-H2AX and TP53 lymphocytes were harvested 12 d after PHA stimulation. Cells were treated either with the appropriate concentration of etoposide or an equivalent volume of dimethyl sulfoxide (DMSO) carrier at 37°C then washed twice in PBS before being fixed in 2% paraformaldehyde and permeablized in PBS 1% Triton X-100. Cells were washed once in PBS 0·1% Triton, resuspended in 500 μl PBS 0·1% Triton and incubated in the presence of 1:1000 dilution of anti-γ-H2AX antibody (ab11174; Abcam, Cambridge, UK) or 1:300 dilution of anti-TP53 antibody (ab32509; Abcam) for 30 min in the dark. Cells were then washed three times in PBS 0·1% Triton, resuspended in 500 μl PBS 0·1% Triton and incubated in the presence of 0·5 μg of Alexa Fluor® 647 goat anti-rabbit IgG (Invitrogen) for 30 min in the dark. Cells were washed three times in PBS 0·1% Triton and resuspended in 300 μl PBS 0·1% Triton + 1 μg/ml 4′,6-diamidino-2-phenylindole (DAPI) to counter-stain nuclei for cell cycle determination before analysis by flow cytometry. Cells were pulse-width gated to exclude doublets.

### Immunocytofluorescence confocal microscopy

Cells were treated either with 5 μmol/l etoposide or an equivalent volume of DMSO carrier for 30 min at 37°C then washed twice in PBS 1% bovine serum albumin before being allowed to gravity settle onto glass slides. Cells were fixed in 4% paraformaldehyde for 15 min and permeablized with 0·5% triton for 10 min. 53BP1 was labelled with a 1/500 dilution of rabbit anti-53BP1 antibody (ab21083; Abcam) and TRF1 was labelled with mouse anti-TRF1 antibody (ab10579; Abcam). Secondary antibodies used were Alexa Fluor® 555 donkey anti-mouse IgG (A-31570; Invitrogen) and Alexa Fluor® 488 goat anti-rabbit IgG (A-11008; Invitrogen). DNA was counterstained with DAPI. Slides were viewed on a Zeiss 510M confocal microscope.

### Statistical analyses

Data were analysed using a Student's *t*-test with *P* values below 0·05 considered significant and below 0·01 considered highly significant. All *P* values are indicated in the text.

## Results

### Patients

The patients in our cohort are detailed in Table I. T lymphocytes were harvested from DC patients’ blood when they attended our clinic. On each occasion T lymphocytes were also acquired from a healthy donor and these were processed and analysed alongside the patient sample to provide paired controls. The purity of the isolated T lymphocytes was determined to be 98·0 ± 1·4% by measuring CD3 expression using flow cytometry. The patients comprised five *DKC1*, six *TERC* and two *TERT* mutations. Several of the *TERC* mutations have previously been assayed for telomerase activity in an *in vitro* fibroblast line system using co-transfection of plasmids bearing *TERT* and *TERC* with the mutations in question and these results have been noted (Table I). There were nine males, four females and the mean age (years) of the group was 25 ± 3 SE (standard error). Of the controls, 10 were male, three were female and the mean age was 37 ± 3 SE. It was not possible to acquire an age- and sex-matched control each time a sample was acquired owing to the availability of suitable controls at the time of the clinic. The telomere length (expressed as Δtel) was determined in the peripheral blood of all but one of the DC patients and in every case telomeres were shorter than normal. Seven were below the first centile and a further four were below the tenth (Table I). All of the patients had one or more abnormal haematological value with the exceptions of patients D2 and D4. Of the 13 DC patients, nine had reduced haemoglobin levels, 10 had reduced platelet counts (and generally were severely affected) and six were lymphopenic. Interestingly, none were T lymphopenic.

**Table I:** Cohort details including mutation status and haematological values

Patient	Affected gene	Gender	Age (years)	Nucleotide mutation (c.)	AA substitution (p.)	Δtel	Telomerase activity (%)	Haemoglobin (g/l)	White cells (×10^9^/l)	Neutrophils (×10^9^/l)	Total lymphocytes (×10^9^/l)	T lymphocytes (×10^9^/l)	Monocytes (×10^9^/l)	Platelets (×10^9^/l)
D1	*DKC1*	M	18	1049T>C	Met350Thr	−1·31	N/A	151	5	3·1	**1·1**	0·8	0·6	**92**
D2	*DKC1*	M	27	1058C>T	Ala353Val	−4·81	N/A	135	6·1	2·1	2·9	2·4	0·7	188
D3	*DKC1*	M	22	1058C>T	Ala353Val	−5·05	N/A	**124**	4·1	2·1	**1·3**	0·9	0·7	**98**
D4	*DKC1*	M	44	1205G>A	Gly402Glu	−3·09	N/A	144	5·9	3·2	1·9	1·1	0·7	213
D5	*DKC1*	M	24	1205G>A	Gly402Glu	−5·34	N/A	142	4·3	2·2	**1·4**	1·0	0·4	187
T1	*TERC*	M	28	2G>C	N/A	−4·82	∼100	**114**	**3·5**	**1·9**	**1·4**	N/E	0·2	**40**
T2	*TERC*	F	26	53_87del	N/A	−2·82	<1	**94**	**2·6**	**1·7**	**0·5**	N/E	0·2	**28**
T3	*TERC*	F	18	110_113del	N/A	−5·32	<1	**116**	7·0	**1·0**	1·7	0·8	0·2	**77**
T4*	*TERC*	M	36	180C>T	N/A	−4·51	∼25	**115**	**3·4**	**1·5**	1·6	1·0	0·2	**38**
T5	*TERC*	F	19	182G>A	N/A	−3·06	∼1	**89**	**2·6**	**1·1**	**1·2**	N/E	0·3	**33**
T6	*TERC*	M	5	178G>A	N/A	−2·16	∼10	**116**	7·1	2·2	3·8	N/E	1·0	**24**
TERT 1	*TERT*	M	17	166G>C	Val56Leu	−3·48	∼4	**66**	**2·6**	**1·0**	N/E	N/E	N/E	**14**
TERT 2	*TERT*	F	27	248G>C	Arg83Pro	N/E	N/E	**75**	5·3	**1·9**	1·9	N/E	0·4	**75**
Normal ranges								135–175	4–11	2–7·5	1·5–4	0·6–2·5	0·2–1	150–400

N/A, not applicable; N/E, value not established. Δtel is the telomere length relative to age-matched healthy controls. Below −3·3 is the first centile, and below −2·1 is the tenth centile. Telomerase activity is the activity relative to wild type enzyme activity as previously assessed in an *in vitro* fibroblast line assay system. Normal ranges for haematological values are indicated below. Where a haematological value is outside of the normal range it is highlighted in bold.

* Patient T4 was under treatment with oxymetholone at the time of this blood count.

### DC lymphocytes have impaired growth characteristics, increased apoptosis and abnormal cell cycle profiles

**Fig 1:**
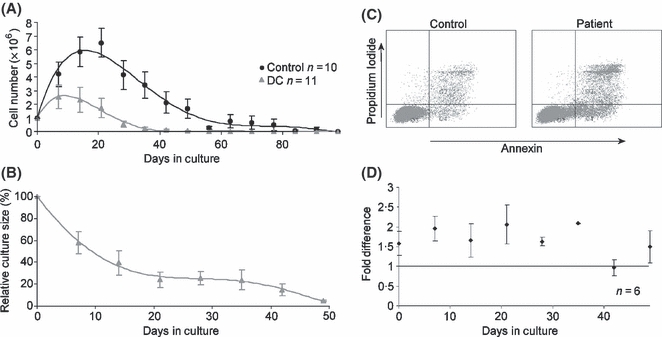
T lymphocytes from DC patients show impaired growth and increased apoptosis. (A) Cell numbers counted every 7 d in T lymphocyte cultures. 1 × 10^6^ cells were plated at day 0 and stimulated with PHA for 48 h. Cells were subsequently cultured in interleukin-2 supplemented medium. (B) DC lymphocyte cell numbers expressed as a percentage of control cell numbers (control *n* = 10) show an increasing growth deficit over the culture period. (C) Propidium iodide and annexin V co-staining of lymphocytes as measured by flow cytometry using sample D1 as an example. On the left is the control sample and on the right the patient sample. (D) A higher proportion of DC lymphocytes (around twofold higher than controls) were apoptotic over 49 d in culture. Error bars represent the standard error from six patient sample measurements (four *TERC*, two *DKC1*) against six paired controls.

**Fig 2:**
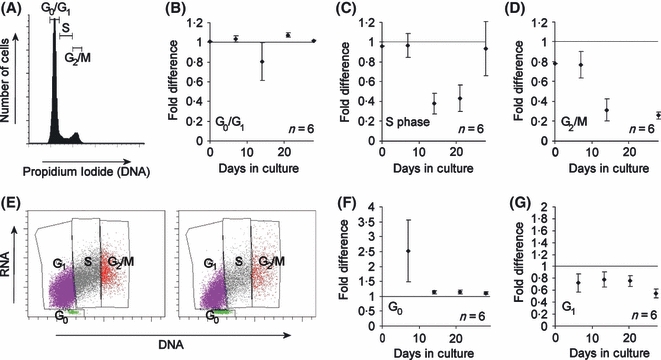
Cell cycle abnormalities in DC lymphocytes after mitogenic stimulation (A) Nuclear staining of lymphocytes with propidium iodide as measured by flow cytometry. The phases of the cell cycle are marked. (B) More DC lymphocytes are in the G_0_/G_1_ phase compared to controls. (C) Fewer DC lymphocytes are in the S phase compared to controls. (D) Fewer DC lymphocytes are in the G_2_/M phase compared to controls. (E) Co-staining of lymphocyte DNA with Hoescht 33342 and RNA with pyronin Y as measured by flow cytometry using sample D1 as an example. On the left is the control and on the right the patient sample. The phases of the cell cycle are marked. (F) More DC lymphocytes are in the G_0_ phase compared to controls. (G) Fewer DC lymphocytes are in the G_1_ phase compared to controls. All graphs express fold difference to control samples. Error bars represent the standard error from six patient sample measurements (four *TERC*, two *DKC1*) against six paired controls.

Despite the fact that none of the patients in our cohort were T lymphopenic, culture of these lymphocytes after stimulation with the mitogen PHA showed a clear and progressive impairment of growth relative to normal controls (Fig 1A, B). This was accompanied by a persistently elevated level of apoptosis (1·5–2-fold higher, *P* < 0·01 by day 28) (Fig 1C, D), a finding that has been demonstrated previously in a single large family with a *TERC* mutation (Knudson *et al*, 2005). Cell cycle analyses of cellular DNA content demonstrated a small but statistically significant increase in the proportion of DC cells in the G_0_/G_1_ phase at days 7 and 21 (*P* < 0·01 at day 21) (Fig 2A, B). In conjunction with this there was a general reduction in the proportion of cells in the S phase (*P* = 0·02 at days 14 and 21) and G_2_/M phase (*P* = 0·02 by day 28) (Fig 2C, D, respectively). A more detailed analysis of cellular DNA and RNA content showed that a higher proportion of DC lymphocytes were in the RNA-low G_0_ phase (*P* = 0·02 by day 28) (Fig 2E–G), consistent with a more senescent population. These data suggest that a combination of increased apoptosis and senescence contribute to growth impairment in DC lymphocytes.

### The DNA damage response is not up-regulated in DC lymphocytes

**Fig 3:**
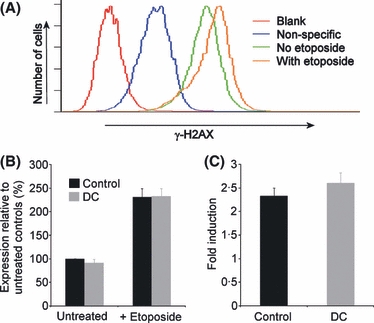
The DNA damage response is not up-regulated in T lymphocytes from our DC cohort. (A) DNA damage associated histone protein γ-H2AX expression as measured by flow cytometry. Fluorescence is measured in the absence of antibodies to determine levels of auto-fluorescence (blank). Fluorescence in the presence of fluorescently-labelled secondary antibody only is regarded as non-specific and is subtracted from all subsequent measurements. Fluorescence in the presence of primary (anti-γ-H2AX) antibody plus secondary is considered specific due to the presence of γ-H2AX. Incubation with etoposide causes a net increase in median fluorescence due to increased activation of H2AX to γ-H2AX. (B) Relative expression of γ-H2AX in DC lymphocytes compared to controls both with and without 350 μmol/l etoposide exposure shows no significant difference. (C) Fold induction of γ-H2AX expression in DC lymphocytes is not significantly different to controls. Error bars represent the standard error from six DC samples (three *TERC*, two *DKC1*, one *TERT*) against six paired controls.

Recent studies have implicated an abnormal DNA damage response in the pathology of DC in fibroblasts from both humans and mouse models (Gu *et al*, 2008; Westin *et al*, 2010). We wanted to determine if this might play a role in the impaired growth phenotype of DC lymphocytes. Previous studies have used phosphorylation of histone protein H2AX to γ-H2AX as an early marker of the cellular response to DNA damage. We therefore measured the levels of γ-H2AX in DC lymphocytes relative to controls using flow cytometry (Fig 3A). Steady state levels were measured along with the level after induction by a 30-min exposure to 350 μmol/l etoposide, a chemical known to cause double-strand DNA breaks (Fig 3B).

The level of steady-state γ-H2AX was not significantly different between controls and DC cells (*P* = 0·31). As expected, exposure to etoposide induced an increase in γ-H2AX expression in both control and DC cells. However, there was no significant difference between controls and DC cells (*P* = 0·88), nor was there a significant difference in the level of induction (i.e. the fold-increase in γ-H2AX level after exposure to etoposide), which was around 2·5-fold in these samples (*P* = 0·35) (Fig 3C).

**Fig 4:**
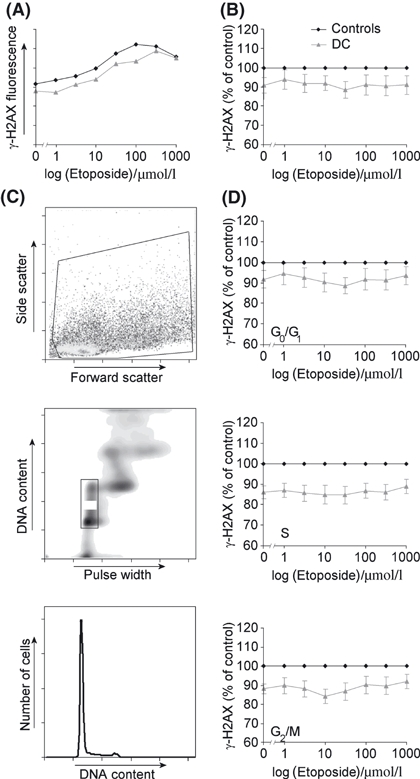
γ-H2AX expression in T lymphocytes assayed in detail across a range of etoposide doses and cell cycle populations. (A) Example of a γ-H2AX dose-response curve for etoposide exposure showing a control sample and a DC sample in the same assay. (B) γ-H2AX expression of nine DC samples (five *TERC*, three *DKC1* and one *TERT*) normalized to seven paired controls. (C) After acquisition of whole cells (upper panel) DAPI staining of the nuclei allows exclusion of doublets (middle panel) and cells in different phases of the cell cycle can be specifically gated (lower panel). (D) Dose-response curves showing γ-H2AX expression in different phases of the cell cycle.

We considered that the concentration of etoposide used might be so high that no discrimination between DC and control would be possible. To determine whether this was the case we repeated the assays using a dose range from 1 μmol/l to 1 mmol/l. As expected, there was a dose-dependent response to etoposide exposure (Fig 4A). The response was broadly similar across the dose range used but, if anything, the level of expression was 10–15%*lower* in the DC lymphocytes, although this did not reach statistical significance (Fig 4B).

We also considered that γ-H2AX expression or etoposide function might be cell-cycle dependent. As cellular DNA was stained with DAPI in this protocol we were able to gate specifically for populations in the G_0_/G_1_, S and G_2_/M phases of the cell cycle (Fig 4C) and repeat the analysis of γ-H2AX expression (Fig 4D). We found that the pattern remained similar for all phases but the reduction in γ-H2AX expression in DC samples was statistically significant in both the S and G_2_/M phases (*P* values <0·03 for all dosages), confirming a cell cycle-dependent effect.

**Fig 5:**
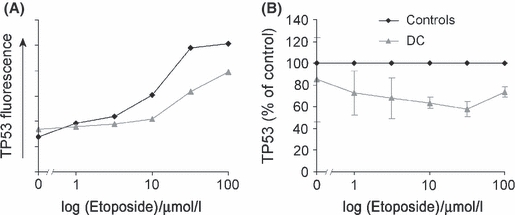
Expression of TP53 in lymphocytes after 6 h of etoposide exposure across a range of concentrations. (A) Example showing that T lymphocytes show a dose-response in TP53 expression after etoposide treatment. (B) There is no increase in TP53 expression in DC cells (one *TERC*, one *TERT*) compared to two paired controls.

The activation of γ-H2AX by DNA damage is part of the ATM-TP53/CDKN1A (p21)-dependent DNA damage checkpoint response (Shiloh, 2003) and TP53 expression has also been shown to be upregulated in mouse embryonic fibroblasts with a *Dkc1* mutation after etoposide treatment (Gu *et al*, 2008). We therefore measured TP53 by flow cytometry after 6 h exposure to etoposide over a range of 1–100 μmol/l. As expected, both DC and control cells showed a dose response (Fig 5A) but again, TP53 levels were, if anything, lower in the DC lymphocytes (Fig 5B), however this was only significant at concentrations of 10 μmol/l and above (*P* < 0·03). These results suggest that the global response to DNA damage is not elevated in DC lymphocytes and in fact may be slightly reduced.

### DC lymphocytes have increased DNA damage at telomeres

**Fig 6:**
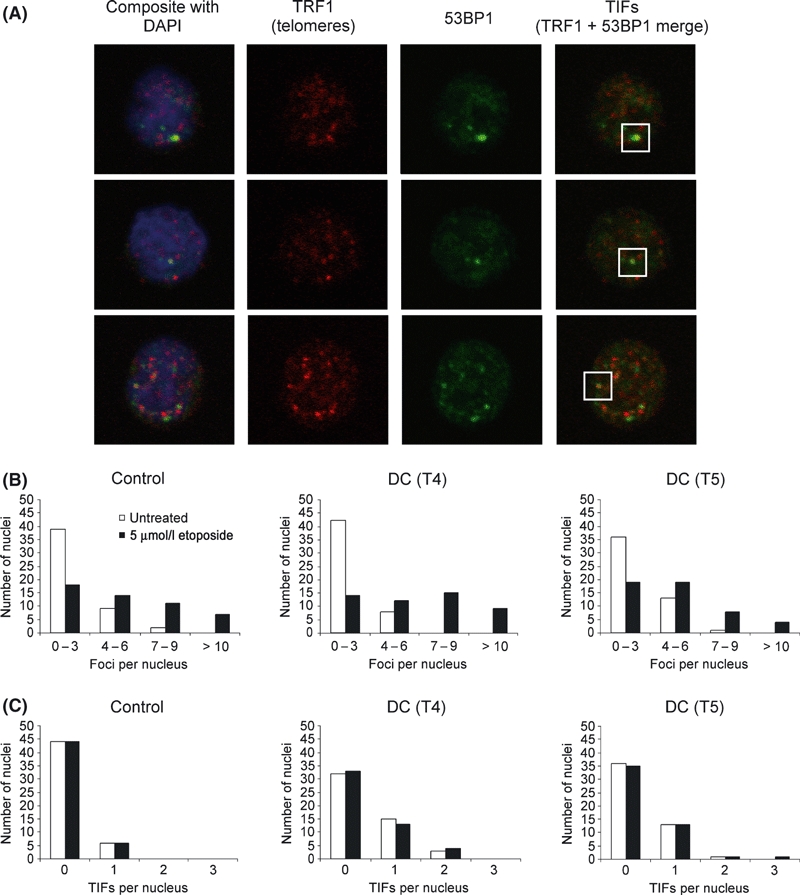
Confocal microscopy showing DNA damage foci in T lymphocytes. (A) Three example images showing nuclei stained with DAPI and labelled for TRF1 telomeric protein, 53BP1 DNA damage protein and a merged image showing 53BP1 colocalizing with telomeres at telomere induced damage foci (TIFs). (B) The number of 53BP1 DNA damage foci per nucleus is similar in a control sample and in two DC samples. (C) There is a small but significant increase in the number of TIFs in the DC lymphocytes. In all cases 50 nuclei were counted.

DNA damage response assays are more usually performed using microscopic quantification of DNA damage foci. To validate our flow cytometry protocol we therefore labelled DC and control cells for 53BP1, a protein known to co-localize with γ-H2AX to sites of DNA damage (Nelms *et al*, 1998) and which gives more easily quantifiable results in microscopic assays (Takai *et al*, 2003) (Fig 6A). Two DC samples were compared to a control sample and 50 nuclei were counted in each. We found no significant difference in the average number of 53BP1 foci per cell between controls and DC patients either with or without etoposide exposure (*P* > 0·1 in all cases) (Fig 6B), consistent with our flow cytometry data obtained with a single etoposide dose.

DNA damage at telomeres has been demonstrated in fibroblasts from both human DC patients and mouse models of the disease (Gu *et al*, 2008; Lamm *et al*, 2009; Touzot *et al*, 2010; Vidal-Cardenas & Greider, 2010). To determine whether this was relevant to DC lymphocytes we co-labelled 53BP1 and the shelterin protein TRF1 and used confocal microscopy to determine the extent of co-localization in DNA damage complexes known as telomere dysfunction-induced foci (TIFs) (Takai *et al*, 2003). Although the number of TIFs was low in both the control and DC samples, there was a significant increase in the number of cells with TIFs and the numbers of TIFs per cell in the DC samples (*P* < 0·01 in both cases) (Fig 6C). Interestingly, exposure to etoposide did not significantly affect the number of TIFs either in control or DC samples (*P* remained below 0·01 vs. control).

These data corroborate the flow cytometry results and confirm that there is no global increase in DNA damage in DC lymphocytes, and no increased response to DNA damaging agents. However, they do demonstrate that there is a small but significant increase in DNA damage at telomeres.

### Steady-state DNA damage is elevated in DC fibroblasts but the DNA damage response is normal

**Fig 7:**
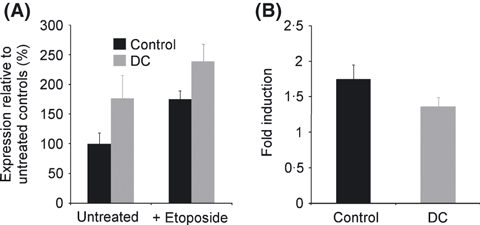
The basal level of DNA damage is increased in DC fibroblasts but the DNA damage response is normal. (A) Relative expression of γ-H2AX in fibroblasts compared to controls both with and without 350 μmol/l etoposide exposure shows a significantly higher level of expression in DC cells. (B) Fold induction of γ-H2AX expression in DC fibroblasts is not significantly different to controls. Error bars represent the standard error from three DC samples (three *DKC1*) against two controls.

As previous DNA damage work has been carried out exclusively on fibroblasts we considered that there might be cell type-specific effects at play. We therefore performed flow cytometric analysis of γ-H2AX in DC fibroblasts. We used a single dose of etoposide because it is difficult to grow sufficient numbers of fibroblasts for a dose-response curve assay. Unlike the T lymphocytes, the DC fibroblasts showed a marked and significant increase in steady-state γ-H2AX level of around 75% compared to controls (Fig 7A) (*P* = 0·04). However after etoposide treatment, although there was still a higher level of γ-H2AX in the DC cells, significance was lost (*P* = 0·31). The level of induction on exposure to etoposide was similar to controls at around 1·5-fold (Fig 7B) and was not significantly different (*P* = 0·08). These data indicate that DC fibroblasts do have more accumulated DNA damage than normal fibroblasts but their response to DNA damaging agents is normal.

## Discussion

This study demonstrated abnormalities in growth, cell cycle profile and DNA damage in cells from DC patients. The cells studied were all genetically unmanipulated, primary tissues and represented three genetic subtypes of the disease.

The majority of published work on DC in humans has suggested that an inability to maintain telomeres, either by a dysfunction in the telomerase enzyme or by structural or mechanistic dysfunction at the telomere itself, results in premature apoptosis or senescence. Indeed, the presence of a short telomere is labelled as DNA damage by the normal cellular response which includes the formation of γ-H2AX- and 53BP1-containing foci (d'Adda di Fagagna *et al*, 2003). However, short telomeres have often not been determined as the primary cause of pathology in studies of mouse models of DC (He *et al*, 2002; Ruggero *et al*, 2003; Mochizuki *et al*, 2004), perhaps highlighting inherent differences in mouse and human cell biology.

Recent work in fibroblasts from both mouse models and human tissues has suggested that an increase in the DNA damage response may contribute to the disease pathology. This has been demonstrated in mouse embryonic fibroblasts with a *Dkc1* mutation by exposure to etoposide (Gu *et al*, 2008). In human fibroblasts with a *TERC* mutation increased DNA damage, similar to that observed in our fibroblasts, was suggested to activate TP53 and thereby sensitize the cells to reactive oxygen species (Westin *et al*, 2010). By studying both fibroblasts and lymphocytes we have shown that an increase in global DNA damage is not a consistent feature of tissues from DC patients. Further, by assessing the level of DNA damage before and after exposure to etoposide we have demonstrated that the response to DNA damage is not elevated and may, in fact, be slightly lower in some DC cells. If cells were continually and excessively activating their DNA damage response then one would also expect an accumulation in the G_2_/M phase as cells that were in the process of growth and division and have accumulated damage foci run up against the G_2_/M checkpoint, a situation commonly observed in lymphocytes from Fanconi anaemia patients (Dutrillaux *et al*, 1982; Kubbies *et al*, 1985; Seyschab *et al*, 1995). Instead we observed a reduction in the S and G_2_/M phase populations and a concomitant increase in the G_0_/G_1_ population consistent with cells containing short telomeres being unable to cycle out of G_1_.

The role of the DNA damage response in DC is not universally agreed upon. One study in *Terc* and *Tert* knockout mouse cells, contrary to other published reports of mouse *Dkc1* mutation, suggested that the number of DNA damage foci and the amount of DNA damage associated protein induction was the same as or lower than in normal mouse cells (Vidal-Cardenas & Greider, 2010). The effect on TP53 regulation is also unclear. While one study observed an increase in TP53 expression in mouse *Dkc1* mutant cells after etoposide exposure (Gu *et al*, 2008), another reported a *decrease*, both in mouse cells and human B lymphocyte lines bearing dyskerin mutations, due to an impairment in internal ribosome entry site (IRES)-mediated TP53 translation (Bellodi *et al*, 2010). Looking back to much earlier work on the exposure of human DC lymphocytes and fibroblasts to clastogens, no significant difference was found compared to normal cells in terms of survival or chromosomal breakage (Dokal *et al*, 1992). If the DNA damage response were abnormally elevated in DC cells then this would affect survival after exposure to DNA damaging agents. However it remains at least possible that, because telomeres are already short in DC patients, the pre-existing disease context could potentially mask some of the effects that are observed in model systems.

Clearly, there is an abnormality in the basal level of DNA damage in the fibroblasts we studied and this is consistent with other published reports. This may well represent an accumulation of damage in cells that would be consistent with the accumulation of senescent fibroblasts with short or dysfunctional telomeres over time in culture. That we did not observe it in lymphocytes suggests that it is not a consistent feature of the disease across tissues. This is in contrast to Fanconi anaemia, in which chromosomal breakage is a consistent feature in both lymphocytes and fibroblasts (Auerbach, 1993). The growth curves for T lymphocytes (Fig 1A) demonstrate a rapid clearance of dead cells from culture, which may explain why such an accumulation does not occur with this cell type. On the other hand, we did observe a small but significant increase in the number of damage foci at telomeres in lymphocytes and again this is consistent with the presence of short or dysfunctional telomeres. Nonetheless, both cell types responded to etoposide exposure in a similar fashion to normal cells, suggesting that their DNA damage response machinery is intact.

While we contend that the role of DNA damage is secondary to telomere shortening in DC, we do not rule out the possibility that it contributes to pathology. Further to this, although features of increased DNA damage response are not displayed in the genetic subtypes of lymphocytes we have studied, additional work is necessary to clarify the situation in other subtypes. In particular, DC caused by mutations in the shelterin component TIN2 would be worthy of particular attention since expression of truncated forms of TIN2 (Kim *et al*, 2004) and inactivation of another shelterin component TRF2 (Takai *et al*, 2003) have been shown to result in the accumulation of γ-H2AX-containing DNA damage foci at telomeres in cell lines. There are also the approximately 50% of genetically uncharacterized cases to consider, whose genetic and molecular mechanisms remain unknown.

Overall, our study points to short or dysfunctional telomeres as the primary cause of pathology in human cases of DC in the context of mutations in *DKC1*, *TERC* and *TERT.* While the accumulation of DNA damage may contribute to the pathology in some tissues, a dysfunctional DNA damage response is unlikely to play a primary role in the overall clinical phenotype in these individuals.
